# Fabrication of Co_3_O_4_/NiCo_2_O_4_ Nanocomposite for Detection of H_2_O_2_ and Dopamine

**DOI:** 10.3390/bios11110452

**Published:** 2021-11-13

**Authors:** Tianjiao Liu, Xiaoyuan Zhang, Kun Fu, Nan Zhou, Jinping Xiong, Zhiqiang Su

**Affiliations:** 1 Beijing Key Laboratory of Advanced Functional Polymer Composites, State Key Laboratory of Chemical Resource Engineering, Beijing University of Chemical Technology, Beijing 100029, China; 2019400133@mail.buct.edu.cn (T.L.); 2020700036@mail.buct.edu.cn (X.Z.); 18811355265@163.com (K.F.); 2021210251@mail.buct.edu.cn (N.Z.); 2 Beijing Key Laboratory of Electrochemical Process and Technology of Materials, Beijing University of Chemical Technology, Beijing 100029, China

**Keywords:** ZIF-67, Co_3_O_4_, NiCo_2_O_4_, electrochemical biosensor, H_2_O_2_, dopamine

## Abstract

Herein, the Co_3_O_4_/NiCo_2_O_4_ nanocomposite has been prepared as a novel electrochemical sensor to accurately detect hydrogen peroxide (H_2_O_2_) and glucose. ZIF-67 is a metal-organic framework (MOF) with Co as the center metal ion. Co_3_O_4_ can be obtained by calcination of ZIF-67 at 700 °C, which can retain the structure of ZIF-67. The hollow Co_3_O_4_ nanocrystal was synthesized based on a calcination process of ZIF-67. This open structure can promote the whole Co_3_O_4_/NiCo_2_O_4_ nanocomposite larger accessible surface area and reactive sites. Co_3_O_4_ has good electrocatalytic performance, which has been applied in many fields. Moreover, H_2_O_2_ and dopamine sensing tests indicate that the as-prepared non-enzymatic electrochemical biosensor has good detection properties. The testing results indicate the as-prepared biosensor has a wide detection range, low detection limit, high selectivity, and long-term stability. These testing results suggest the potential application in food security, biomedicine, environmental detection, and pharmaceutical analysis.

## 1. Introduction

Many biomolecules play an essential role in vital activities and biochemical reactions in the human body, such as transmitting biological molecules and responses in natural metabolism [1]. Common biological molecules are glucose [2], uric acid [3], RNA [4], ascorbic acid, hydrogen peroxide (H_2_O_2_) [5], and dopamine [6,7]. The existence of these substances can maintain the normal physiological activities of the human body. However, once the concentration of these substances is too low or too high, it may cause various diseases. For example, dopamine, produced by the brain, is a typical small biological molecule in the human body to control the emotion of humans. Once the concentration is too high, people may get schizophrenia. On the contrary, if dopamine concentration in the human body is too low, people may get depression [6,7]. H_2_O_2_ concentration in some parts of the human body is also an important healthy index, which if sustained in a normal range, can keep human health [5,8,9]; therefore, it is very important to detect biological small molecules rapidly and accurately for disease diagnosis and clinic treatment [10,11]. The current methods for detecting these small biological molecules in vivo include fluorescence spectrometry, colorimetric analysis, high-performance liquid chromatography, and electrochemical analysis [12,13].

The electrochemical analysis is usually to establish an electrochemical system. The detected small biological molecules are used as a part of this system, and then the relationship between the current of the system and the concentration of the detected substances is analyzed. This method is named the electrochemical biosensor. Compared to other methods, electrochemical biosensors have low cost, a small volume, an easy to build system, good selectivity, high sensitivity, a fast response, good biocompatibility, and many other advantages that have attracted the attention of every corner of the world [14,15,16]. Recent research has reported 3D porous reduced graphene oxide (RGO) decorated with MoS_2_ quantum dots to detect H_2_O_2_. RGO can be used as an electrochemical biosensor to accelerate the ion transform of the reaction system, to improve the electrical property [17]. Another researcher displayed a CuO/PANI hollow nanofiber to detect H_2_O_2_ and glucose simultaneously. This hollow fiber can provide a more specific surface area, offering more active reaction sites to electrochemical reactions [18]. In addition, there are many other materials used in electrochemical biosensors, which will not be described in this paper.

Metal-organic framework (MOF) is a novel material developed in recent years [19,20]. It forms intramolecular pores by self-assembling organic ligands and metal ions or clusters through coordination bonds. When changing the carbon chain length, the structures and pore sizes can be adjusted [21], and different center metal ions can be introduced to MOF materials with various structures due to other self-assembly methods of different metal ions. Similarly, different functional groups and ligands can also lead to MOF materials with different properties [22,23,24,25]. MOF has adjustable pores, an ordered crystal structure, and large specific surface areas widely used in electrocatalysis, gas adsorption, energy storage, and electrochemical sensing [26].

MOF materials can be synthesized by solution, ultrasonic, microwave, diffusion, and hot solvent methods [26]. Among these methods, the solution method is the most common method for synthesizing MOF, a simple stirring process that can produce many MOF materials. This preparation method is also used in this paper. MOF synthesized by the water thermal method generally has two valences of metal ions in these MOF materials. Therefore, the water thermal method mainly focuses on obtaining homogeneous MOF crystals.

ZIF-67 is a kind of hollow polyhedral material and expresses high thermal stability and chemical robustness. Although this structure allows MOF to have a suitable electrochemical property, the conductive property of MOF is not as good. MOF materials are often used in combination with other materials; the doped materials can possess the properties of both materials. The properties of these materials have been improved in all aspects. NiCo_2_O_4_ is a layered double oxide material with a spinel structure. Generally, NiCo_2_O_4_ is synthesized by a simple water-thermal method. The morphology of NiCo_2_O_4_ can be control through different reaction conditions. NiCo_2_O_4_ has many advantages for electrode material, such as cheap, environment-friendly, good electrical conductivity, and abundant REDOX site on the surface. These properties can lead NiCo_2_O_4_ to be more used in modified composite materials to obtain novel materials with improved materials.

**Figure 1:**
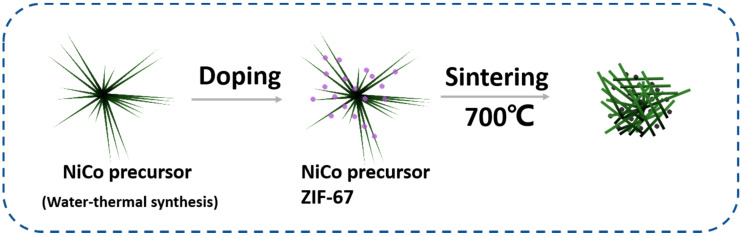
Schematic description for synthesizing Co_3_O_4_/NiCo_2_O_4_ with high-temperature calcination of the ZIF-67/Ni-Co composite.

In this paper, we proposed a novel Co_3_O_4_/NiCo_2_O_4_ composite. Hollow Co_3_O_4_ nanocrystal was obtained by ZIF-67 calcinating at high temperature, and the production had retained the regular dodecahedron structure ZIF-67 possesses. The as-prepared Co_3_O_4_ has excellent electrochemical properties in electrocatalysis, rechargeable batteries, and electrochemical biosensors. NiCo_2_O_4_ was formed in the nanorod structure, connecting the scattered Co_3_O_4_ nanocubes to generate Co_3_O_4_/ NiCo_2_O_4_ composite, which improved electrical property. The as-synthesized material was utilized as the electrochemical biosensors expressed a low detection limit and wide detected range compared to similar biosensors. These properties of as-prepared Co_3_O_4_/ NiCo_2_O_4_ composite mainly have two advantages in sensing. At first aspect, Co_3_O_4_/ NiCo_2_O_4_ composite has high specific surface area, which can provide more reaction active sites to electrochemical reaction. On the other hand, Co_3_O_4_/ NiCo_2_O_4_ composite contain the elements favorable for electrochemical biosensor. When the as-prepared biosensor was used to detect other biomolecules, such as glucose, urea acid, and KCl, the curve had almost no response, which demonstrated that the as-prepared biosensor had good selectivity to H_2_O_2_ and dopamine. The synthesized process and detection mechanism are provided in Figure 1.

## 2. Materials and Methods

### 2.1. Materials

Cobalt nitrate hexahydrate (Co(NO_3_)_2_·6H_2_O), cetyl trimethyl ammonium bromide (CTAB, 99%), phosphate balances normal saline (PBS 1M pH = 7.2–7.4), dopamine (DA, 98%), potassium chloride (KCl, 99.5%), uric acid (UA, 98%) and urea (99%) were purchased from J&K (Beijing, China). Hydrogen peroxide (H_2_O_2_, 30%) was purchased from Beijing Chemical Co. Ltd. (Beijing, China), glucose was purchased from Macklin Biochemical Co. Ltd. (Shanghai, China). Cobalt chloride (CoCl_2_, 98%) and nickel chloride (NiCl_2_, 98%) were purchased from Energy Chemical Co., Ltd. (Shanghai, China). 2-methylimidazole and Nafion (5%) were purchased from Sigma-Aldrich (Shanghai, China). Methanol was purchased from Fuchen Chemical Co., Ltd. The water was purified through a Millipore system (≈18.2 MΩ cm, Zhongyang Technology Development Co., Ltd., Beijing, China).

### 2.2. Synthesis of Ni-Co Precursor

The Ni-Co precursor was synthesized by a hydrothermal synthesis method. At first, CoCl_2_ (0.1 mol), NiCl_2_ (0.05 mol), CTAB (0.04 mol), and urea (0.18 mol) were mixed in moderate deionized (DI) water and stirring for 30 min until all substances were fully dissolved and evenly mixed. Then, the as-prepared light red transparent solution was put in a Teflon-lined container and heated at 100 °C for 12 h. When the reaction finished, the container was taken out after cooling. Next, we centrifuged the reaction solution, collected the precipitation, and washed it three times with DI water and ethyl alcohol. Lastly, the residue was dried in a vacuum oven overnight.

### 2.3. Synthesis of Co_3_O_4_/NiCo_2_O_4_

To synthesize Co_3_O_4_/NiCo_2_O_4_, we synthesized ZIF-67/Ni-Co composite firstly. The as-prepared Ni-Co precursor was immersed in a Co(NO_3_)_2_ (1 mmol) methanol solution, while the Ni-Co precursor concentration in methanol solution was 1 mmol. 4 mmol 2-methylimidazole was put in a 250 mL beaker, we added methanol, and poured the 2-methylimidazole methanol solution into the former solution after 2-methylimidazole was fully dissolved in methanol. The mixed solution was stirring for 30 min and centrifugated after being aged for 24 h.

### 2.4. Preparation of Electrochemical Biosensor and Electrochemical Test

A three electrodes system synthesized the electrochemical biosensor. In this electrochemical system, a saturated amalgam electrode was used as a reference electrode, a platinum foil electrode was utilized as an auxiliary electrode, and a working electrode was a self-made electrode. Before synthesizing the working electrode, the glass carbon electrode (GCE) had to be polished using 3.0 µm, 0.1 µm, and 0.05 µm alumina polishing powder for 10 min and washed by ethanol dried in the air, respectively. This three-electrode system performed electrochemical tests. The cyclic voltammetry (CV) curve and I-t curve were measured with a CHI760E electrochemical workstation (Shanghai, China). A PBS solution (0.1 M pH = 7.2–7.4) was used as the electrolyte in all electrochemical tests. Before the electrochemical tests, the PBS solution was fully deoxygenated by nitrogen. All of the tests were carried out under atmospheric conditions, at room temperature.

## 3. Results and Discussion

### 3.1. Characterization of Co_3_O_4_/NiCo_2_O_4_

**Figure 2:**
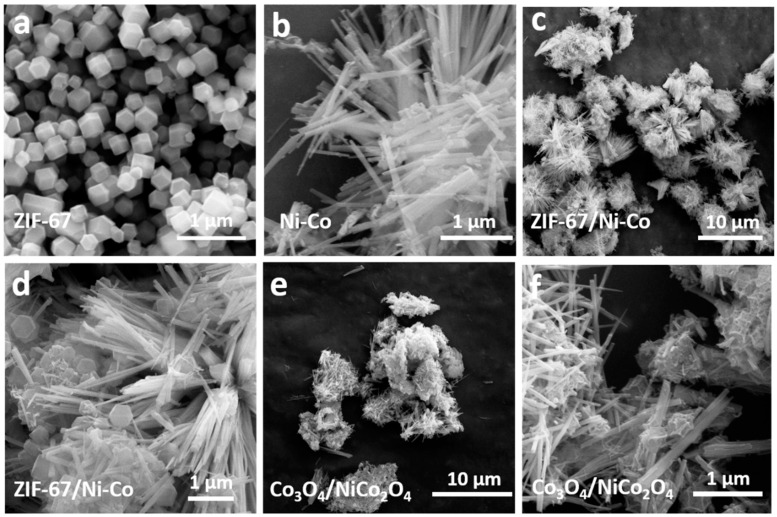
SEM images of the (**a**) ZIF-67 nanocrystal, (**b**) Ni-Co nanorod precursor, (**c**,**d**) ZIF-67/Ni-Co nanocomposite, and (**e**,**f**) Co_3_O_4_/NiCo_2_O_4_ nanocomposite, in different magnifications.

In this part, some analysis and test methods were used to characterize Co_3_O_4_/NiCo_2_O_4_ and a series of intermedia products, during the whole preparation process. A scanning electron microscope (SEM, JEOL, S4700, Beijing, China) was used to observe the surface morphology of materials. ZIF-67 crystal was synthesized by the solution precipitation method, as exhibited in Figure 2a; the size scale was around 200–300 nm. The size of ZIF-67 was generally uniform, and the ZIF-67 crystals had a dodecahedron appearance. It was purple to the naked eye. The following intermedia product was Ni-Co nanorods, the precursor material of the NiCo_2_O_4_ nanorod. The morphology of the Ni-Co nanorod is displayed in Figure 2b. Ni-Co precursor was mainly displayed as nanorod morphology, and the diameter of these rods was around 50–100 nm. Moreover, the Ni-Co nanorod was immersed in Co(NO_3_)_2_ methanol solution to absorb Co^2+^; 2-methylimidazole was added in the former solution to synthesize ZIF-67 on the surface of Ni-Co nanorod. The morphology of ZIF-67/Ni-Co nanocomposite is displayed in Figure 2c–f demonstrate that the morphology of Co_3_O_4_/NiCo_2_O_4_ is a nanocomposite. The morphology of production did not change much compared to the reactants. After calcination at 700 °C, the previously slippery surface of ZIF-67 contracted and formed many folds.

**Figure 3:**
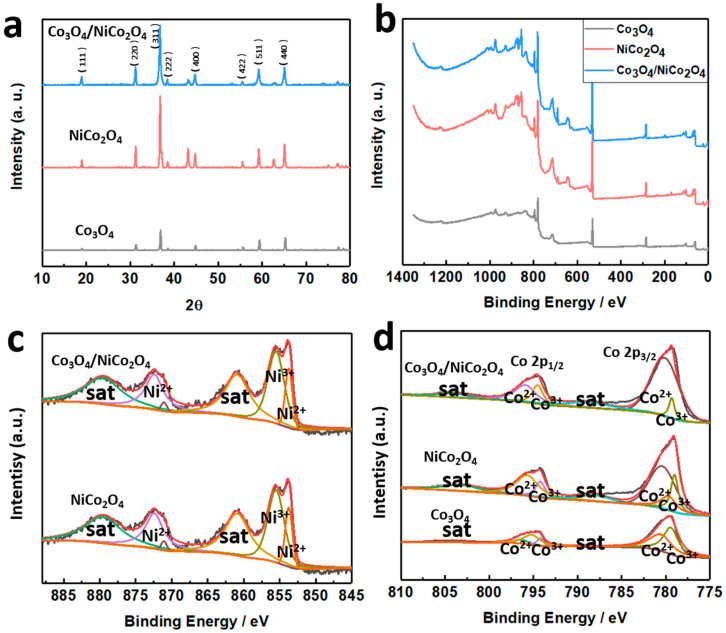
(**a**) XRD spectrum of Co_3_O_4_, NiCo_2_O_4_, and Co_3_O_4_/NiCo_2_O_4_, (**b**) XPS of Co_3_O_4_, NiCo_2_O_4_, and Co_3_O_4_/NiCo_2_O_4_, (**c**) the result of Ni 2p, and (**d**) Co 2p.

X-ray diffraction (XRD, Bruker, Ultima IV X) spectrum and X-ray photoelectron spectroscopy (XPS, EDAX, UX-2500MA) were used to characterize productions that occurred during the preparation process, and the test results are displayed in Figure 3. Figure 3a displays the XRD spectrum of Co_3_O_4_, NiCo_2_O_4_, and Co_3_O_4_/NiCo_2_O_4_. The peaks that occurred in Figure 3a conformed to the standard PDF card of NiCo_2_O_4_ (PDF#20-0781) and Co_3_O_4_ (PDF#42-1467). This indicated that the products could be confirmed to be Co_3_O_4_, NiCo_2_O_4_, and Co_3_O_4_/NiCo_2_O_4_. Figure 3b displayed the XPS image of Co_3_O_4_, NiCo_2_O_4_, and Co_3_O_4_/NiCo_2_O_4_, and Figure 3c,d displayed the XPS images of Ni 2p and Co 2p, respectively. The XPS images demonstrated the elements found and the surface chemical condition of substances. A C 1s, peak at 284.28 eV, was used to calibrate all peaks in this article. There were two 2p spin orbits of Ni^2+^ and Ni^3+^ in Figure 3c. The fitting peak 872.7 eV represented Ni 2p_1/2_, which can be divided into 872.7 eV and 870.8 eV, with a satellite shake-up peak of 879.2 eV. These two peaks represented Ni^3+^ and Ni^2+^, respectively [27]. Similarly, there were fitting peaks at 855.3 eV (Ni^3+^) and 853.6 eV (Ni^2+^) and a satellite shake-up peak of 860.8 eV. These peaks can be found both on Co_3_O_4_/NiCo_2_O_4_ and NiCo_2_O_4_ curves, which can prove the existence of these two substances. The analysis of Co was the same as Ni in Figure 3d. There were two 2p spin orbits of Co^2+^ and Co^3+^, and the fitting peaks were at 794.9 eV and 784.5 eV, which were Co 2p_1/2_ and Co 2p_3/2_. There were also two satellite shake-up peaks at 804.1 eV and 788.6 eV, respectively [28]. 

### 3.2. Electrochemical Tests

**Figure 4:**
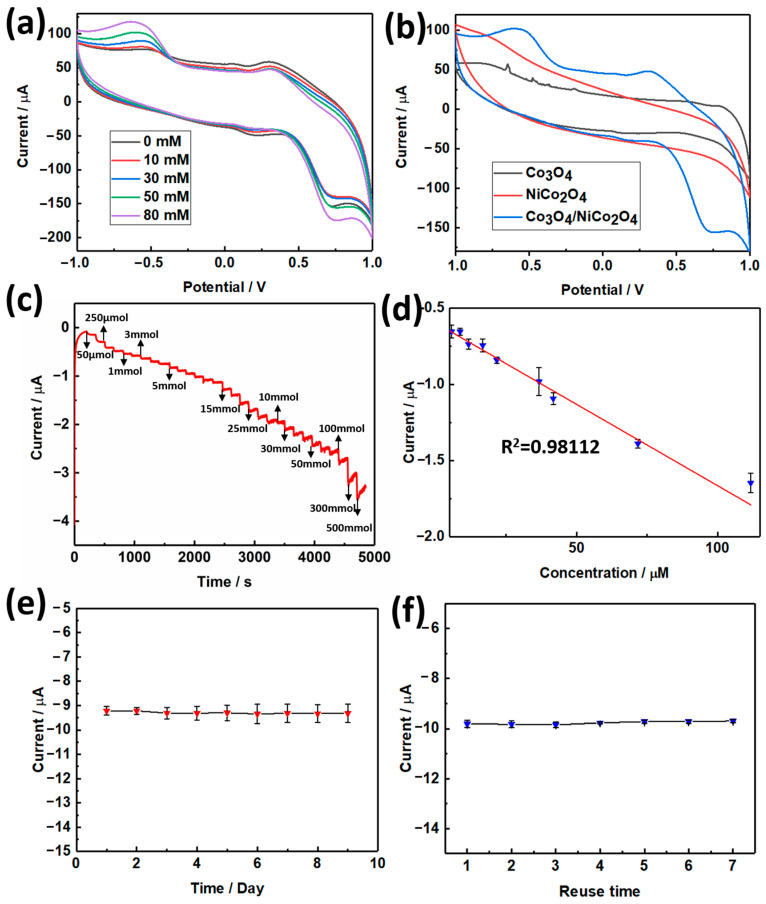
(**a**) The CV of Co_3_O_4_/NiCo_2_O_4_ at 50 mV/s, adding H_2_O_2_ in different concentrations. (**b**) The CV of Co_3_O_4_, NiCo_2_O_4_, and Co_3_O_4_/NiCo_2_O_4_, with a scanning rate of 50 mV/s. (**c**) The I-t curve of Co_3_O_4_/NiCo_2_O_4_ when H_2_O_2_ was added in different concentrations every 150 s. (**d**) The linear regression curve, drawn according to Figure 4c. (**e**) The long time service life test of the H_2_O_2_ electrochemical biosensor. (**f**) The reuse time test of the H_2_O_2_ electrochemical biosensor.

The as-prepared materials were used to fabricate electrochemical biosensors to detect hydrogen peroxide (H_2_O_2_) and dopamine. A three-electrode system established the biosensor. In this system, the self-made Co_3_O_4_/NiCo_2_O_4_ electrode was used as a working electrode. The method of fabricating a working electrode was introduced in Section 2.4. When the three electrodes system was set up, we first used it to test the CV curve. The potential range was set as −1 to 1 V, and the scanning rate was 50 mV/s. The results of CVs are displayed in Figure 4. Figure 4a demonstrated the CV curve with H_2_O_2_ added in different concentrations. The oxidation-reduction peak was enhanced as the concentration of H_2_O_2_ increased, which demonstrated the effect of H_2_O_2_ on the electrochemical system. Figure 4b displays the CV curve of Co_3_O_4_, NiCo_2_O_4_, and Co_3_O_4_/NiCo_2_O_4_, with the scanning rate at 50 mV/s. Then, the I-t curve was tested based on the three-electrode system; the applied potential was 0.3 V, the sampling interval was 150 s. When H_2_O_2_ was added to the electrochemical system, the current value suddenly decreased, and the higher the H_2_O_2_ concentration added, the lower the current was, as displayed in Figure 4c. Figure 4d displays the linear regression curve, drawn with the added concentration and corresponding current. The relationship of added H_2_O_2_ concentration and corresponding current can be fitted to a line, and the R^2^ of this line was equal to 0.98112. The linear regression equation of H_2_O_2_ detection was y = −0.01068x − 0.59764. According to this curve, the limit of detection (LOD) can be calculated as 0.2587 µM (S/N = 3), and the detection range was 0.05–41.7 mM. As an electrochemical biosensor, it must possess as much cyclic time use as possible; Figure 4e,f illustrate the longest cyclic and reuse time of the Co_3_O_4_/NiCo_2_O_4_ electrochemical biosensor. The CV test of the Co_3_O_4_/NiCo_2_O_4_ electrode was tested under the condition of adding 50 mM H_2_O_2_.The results displayed that a self-made Co_3_O_4_/NiCo_2_O_4_ electrochemical biosensor can retain current at a stable condition for nine days and can be reused at least seven times.

Compared to other similar electrochemical biosensors, the as-prepared self-made Co_3_O_4_/NiCo_2_O_4_ electrochemical biosensor has advantages. The parameters of various electrochemical biosensors are displayed in Table 1, which indicates that the self-made Co_3_O_4_/NiCo_2_O_4_ electrochemical biosensor had a wide electrochemical window and low LOD compared with a similar H_2_O_2_ electrochemical biosensors, which meant that the sensor has high competitiveness in the same sensors.

**Figure 5:**
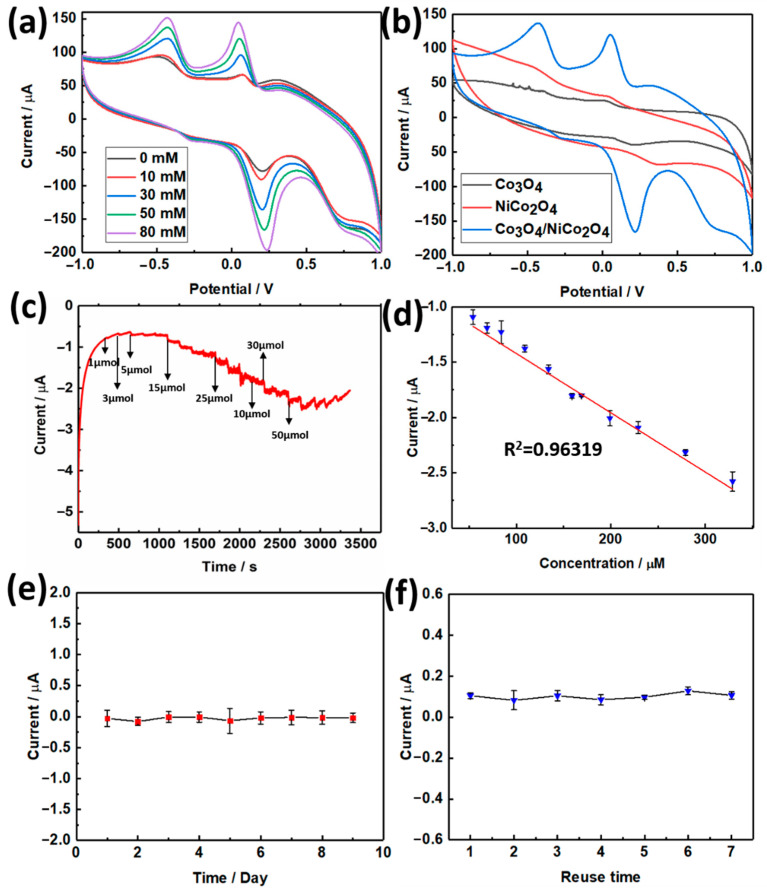
(**a**) The CVs curve of the Co_3_O_4_/NiCo_2_O_4_ electrochemical biosensor, detecting dopamine with different concentrations at a scanning rate of 50 mV/s. (**b**) The CVs curve of Co_3_O_4_, NiCo_2_O_4_, Co_3_O_4_/NiCo_2_O_4_ at a scanning rate of 50 mV/s, with the addition of 50 µM of dopamine. (**c**) The I-t curve of the Co_3_O_4_/NiCo_2_O_4_ electrochemical biosensor, adding different concentrations of dopamine. (**d**) The linear regression curve, drawn based on Figure 3c. (**e**) The long time service life test of the dopamine electrochemical biosensor. (**f**) Th reuse times test of the dopamine electrochemical biosensor.

Besides detecting H_2_O_2_, a self-made Co_3_O_4_/NiCo_2_O_4_ electrochemical biosensor can also play a role in detecting dopamine. The dopamine electrochemical biosensor experiments were tested by the three-electrode systems mentioned before, and the result of the electrochemical test are displayed in Figure 5. The CV curve of the Co_3_O_4_/NiCo_2_O_4_ electrochemical biosensor, adding different dopamine concentrations at a scanning rate of 50 mV/s, is displayed in Figure 5a. When the concentration increased, the oxidation-reduction peaks strengthened. The addition of dopamine reacted with active substances in the electrochemical system. Dopamine has two hydroxyl groups in its benzene ring group; when dopamine is added in the electrochemical system, the Co_3_O_4_/NiCo_2_O_4_ in the electrode oxidize these hydroxyl groups to form quinones, and then quinones are reduced to phenols. Figure 5b displays the CV curves of Co_3_O_4_, NiCo_2_O_4_, and Co_3_O_4_/NiCo_2_O_4_ with 50 mM of dopamine, at a scanning rate of 50 mV/s. The I-t curve of Co_3_O_4_/NiCo_2_O_4_ was used to study dopamine’s detection effect, and the result is presented in Figure 5c; the applied potential was 0.3 V and the sampling interval was 150 s. With the adding of dopamine, there was a sudden change in current values. As the concentration increased, the present change increased, which meant a stronger current response. When the I-t test finished, we drew the curve of linear regression curve using current as the Y-axis and adding dopamine concentration as the X-axis, as demonstrated in Figure 5d with R^2^ = 0.96315. The linear regression equation of dopamine was y = −0.00535x − 0.8867. The detection range of the Co_3_O_4_/NiCo_2_O_4_ electrochemical dopamine biosensor was 24–329 µM. The LOD was calculated to be 0.2410 µM.

Similarly, the long cyclic and reuse time of the electrochemical dopamine biosensor were also tested, as demonstrated in Figure 5d,e. The results in Figure 5e indicate that the electrochemical dopamine biosensor could retain a certain level of initial current value after 15 days, and the dopamine biosensor can be reused eight times, and sustain the initial current, which means the electrochemical dopamine biosensor can be used at least eight times.

Table 2 exhibits several similar electrochemical dopamine biosensors. The as-prepared electrochemical dopamine biosensor was wide in the electrochemical dopamine detection range and low in the detection limitation, compared with similar electrochemical dopamine biosensors. Thus, the as-prepared dopamine electrochemical biosensor had superiority over these similar sensors.

**Figure 6:**
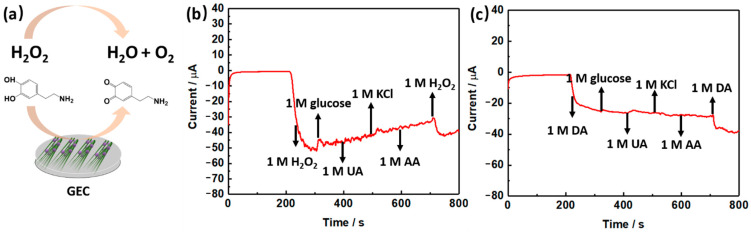
(**a**) The mechanism of electrochemical biosensor detection of H_2_O_2_ and dopamine. (**b**) The selective ability test of the Co_3_O_4_/NiCo_2_O_4_ electrochemical biosensor to H_2_O_2_ and (**c**) DA.

The mechanism of the electrochemical biosensor is displayed in Figure 6a. The electrochemical biosensor can react with tested materials and produce an electrical signal proportional to the concentration of tested materials. When H_2_O_2_ responded with active electrode materials, H_2_O_2_ would transform to H_2_O and O_2_. This process would produce a current signal, which was enhanced with increased concentration. The whole reaction process occurred on the surface of the electrode in PBS solution as the electrolyte. Similar to H_2_O_2_, when dopamine is in contact with the electrode surface, the electrochemical system generates another current signal. The oxidation reaction of catechol to o-quinone in DA occurred, causing the current signal. These processes are exhibited in Figure 6a. Then, to confirm the selectivity of the Co_3_O_4_/NiCo_2_O_4_ electrochemical biosensor, we chose some small bio-substances, such as glucose, UA, KCl, and AA, as interfering substances with H_2_O_2_ and dopamine to test the same condition. Figure 6b,c demonstrate that only dopamine and H_2_O_2_ impact the curve, and other substances have little effect on the curve. This means the self-made electrochemical biosensor has high selectivity to H_2_O_2_ and dopamine and is undisturbed by other substances.

## 4. Conclusions

In summary, a hollow Co_3_O_4_ nanocrystal was established based on the NiCo_2_O_4_ nanorod through simple hydrothermal synthesis and a high-temperature calcination method. The NiCo_2_O_4_ nanorod had more free electrons and holes. The structure of the nanorod can reduce polarization. Moreover, the specific surface area of the rod-like structure of NiCo_2_O_4_ was more significant than similar materials. It improved the active sites of a redox reaction, which proved the electrochemical properties of a self-made electrochemical biosensor.

Furthermore, a Co_3_O_4_ nanocrystal, using ZIF-67 as a matrix, was doped in the NiCo_2_O_4_ nanorod, which also facilitated the properties of the as-prepared nanocomposite. The as-synthesized material next was utilized to fabricate the electrochemical biosensor, to detect H_2_O_2_ and dopamine. The Co_3_O_4_/NiCo_2_O_4_ biosensor can detect H_2_O_2_ and dopamine simultaneously and has no response with other substances, which illustrates the selectivity of the self-made Co_3_O_4_/NiCo_2_O_4_ biosensor. More importantly, the self-made Co_3_O_4_/NiCo_2_O_4_ biosensor had an extensive detection range and low detection limit compared to similar biosensors. When detecting H_2_O_2_, the detection range was 0.05–41.7 mM, the detection limit was 0.2587 µM, and the detection range of dopamine was 24–329 µM, with the detection limit of 0.2410 µM. These results also indicate that the self-made Co_3_O_4_/NiCo_2_O_4_ sensors have potential application prospects in future electrocatalysis and electrochemical devices.

## Figures and Tables

**Table 1:** Comparison of the performance of several different H_2_O_2_ electrochemical sensors.

Materials	Linear Range (mM)	LOD (µM)	Ref.
MnO_2_-ERGO paper	0.1–45.4	10	[29]
N-doped hollow carbon sphere (N-HCS)	0.05–47.5	20	[30]
c-ZnO nanosheets	0.001–10	0.8	[31]
Au/Co_3_O_4_-CeOx nanocomposites	0.01–1	5.29	[32]
Co_3_O_4_/MWCNTs/gelatin/HRP	0.74–19	0.74	[33]
AgNFs-Pt@BSA/GA/GOD	1–14	300	[34]
v-AuNWs/PDMS	0.04–15	12	[35]
Co_3_O_4_/NiCo_2_O_4_	0.05–41.7	0.2578	This work

**Table 2:** Comparison of the performance of several different dopamine electrochemical sensors.

Materials	Linear Range (µM)	LOD (µM)	Ref.
Au-MEA/PEDOT-Tyr	20–300	0.24	[36]
GO/P(ANI-co-THI)	2–500	2	[37]
PA-MNPs/GCE	100–900	7.25	[38]
H-GO/GCE	0.5–40	0.17	[39]
Fe_2_O_3_–NG/GCE	0.5–10; 10–400	0.08	[40]
IL G	0.679	0.679	[41]
IDEs-SV	0.05–0.25	0.6	[42]
Co_3_O_4_/NiCo_2_O_4_	24–329	0.2410	This work

## Data Availability

Not applicable.
